# To stop or not to stop: the clinical dilemma of long-term double-seronegative neuromyelitis optica therapy *

**DOI:** 10.1055/s-0045-1814372

**Published:** 2026-02-04

**Authors:** Tarso Adoni

**Affiliations:** 1 Universidade de São Paulo, Faculdade de Medicina, Departamento de Neurologia, São Paulo SP, Brazil.

Double-seronegative neuromyelitis optica spectrum disorder (DS-NMOSD) is a heterogeneous condition, with patient outcomes ranging from a monophasic episode in about one third of cases to recurrent and often severe attacks. 1 While the presence of serum anti-aquaporin-4 (anti-AQP4) antibodies after a single episode warrants immediate immunosuppressive treatment, this decision is far less clear for DS-NMOSD, according to the 2015 diagnostic criteria of the International Panel for Neuromyelitis Optica Diagnosis (IPND), 2 regardless of disease course. Given that immunosuppressive therapy carries risks such as increased infections and reduced immune surveillance against tumors, deciding to initiate or discontinue treatment in the face of an uncertain risk of recurrence remains a significant challenge in clinical practice. 3

Although the phenotypic presentation of NMOSD is often indistinguishable between groups, some studies suggest genetic and biomarker differences. 4 A key issue is the lack of a reliable marker to guide the optimal therapeutic approach for DS-NMOSD. Furthermore, the underlying pathophysiological mechanism of the double-seronegative forms remains unclear, and it is questionable whether their similarities with anti-AQP4-positive NMOSD extend beyond their clinical presentation. 5 This is supported by data from clinical trials with drugs such as inebilizumab and satralizumab, which showed better disease control in seropositive patients, indicating that the same drugs may not provide the same therapeutic benefit to both groups. 6 7 8

The debate over treatment discontinuation highlights a core disagreement. In the current issue of *Arquivos de Neuro-Psiquiatria* , Silva 9 argues for a rational, individualized approach. I would add that we need to keep in mind that the current controversy exists because the discussion about treatment discontinuation is based exclusively on anecdotal reports and retrospective data. Silva points out that, in addition to the risks of long-term medication, economic and psychological factors must be considered. In carefully selected cases, discontinuing treatment after 10 years of stability could be a reasonable option, with the decision always being shared with the patient. Some small, observational studies also seem to indicate that the longer the period without relapses in double-seronegative patients, the greater the chance of disease remission after discontinuation of immunosuppressive treatment.

On the other side, Pimentel and Becker 10 maintain that the risk of an NMOSD relapse, even in DS-NMOSD, is too great to warrant treatment discontinuation. They emphasize that while reports of late relapses in anti-AQP4-positive NMOSD cannot be directly extrapolated, the potential for severe disability means treatment should only be interrupted in exceptional circumstances. Even then, the decision should only be made if there is a strong desire initially expressed by the patient.

It is important to emphasize that each case of DS-NMOSD should be seen as a syndrome and not as a specific disease and that the physician should always ensure that the anti-aquaporin 4 (AQP4) and anti-myelin oligodendrocyte glycoprotein (MOG) antibodies were screened by the gold standard, the live cell-based assays (CBA) method. Therefore, periodic differential diagnostic evaluation is crucial to rule out other conditions such as sarcoidosis, spinal dural fistula, spinal cord infarction, Behçet's disease, lymphoma, syphilis, and Susac.

In conclusion, the decision to discontinue treatment for DS-NMOSD patients after a decade of stability remains highly complex. Given the lack of definitive data, there is no single, standardized recommendation. The choice must be based on a thorough, individualized analysis of each case, with the patient and clinician navigating a landscape of uncertain variables together. The scientific community urgently needs high-quality studies that include validated biomarkers and reliable risk stratification tools that can allow for an individualized, evidence-based approach before any action is taken regarding the treatment management of this particular group of patients.
